# Idiopathic spontaneous rupture of unscarred uterus in a primigravida in active labour

**DOI:** 10.1016/j.ijscr.2022.107749

**Published:** 2022-10-13

**Authors:** Mona Mishra, Y.M. Mala

**Affiliations:** Department of Obstetrics and Gynecology, Maulana Azad Medical college and Lok Nayak Hospital, New Delhi, India

**Keywords:** Idiopathic, Rupture, Unscarred uterus, Primigravida, Case report

## Abstract

**Introduction:**

Rupture of unscarred pregnant uterus is a rare occurrence and its incidence is higher in developing countries. Rupture of unscarred uterus is associated higher likelihood of adverse maternal and fetal outcomes. Occurrence of rupture of an unscarred uterus lays foundation for the importance of supervised labour and to maintain a high index of suspicion even in a prim gravida.

**Case report:**

We present a case of spontaneous rupture of unscarred uterus in a primigravida with no known risk factors. Rupture was diagnosed in second stage of labour when there was cessation of contractions and loss of station. On exploratory laparotomy, hemoperitoneum of 100 ml and a 10 cm tear was found in left posterolateral aspect of uterus. The tear was repaired successfully and patient had a normal post- operative course.

**Discussion:**

Presence of a uterine scar is the key factor leading to rupture. Spontaneous rupture of unscarred uterus is a rare entity and is associated with multiple factors. In our case, all these factors were ruled out. In literature search we could find 15 cases of spontaneous rupture in unscarred uterus. According to our best knowledge this is the 7th case of rupture in unscarred uterus, reported in a prim gravida without any obvious risk factors.

**Conclusion:**

Rupture uterus should be kept in mind in all patients, even in primigravida if there is high index of suspicion. Quick diagnosis and immediate laparotomy is the cornerstone of treatment in such cases.

## Highlights

• Rupture of unscarred uterus is rare but is associated with significant morbidity.
• High index of suspicion is needed even in absence of risk factors.
• Immediate laparotomy is the gold standard management.
• Delay in diagnosis and management can lead foeto-maternal morbidity.

## 1 Introduction and importance

Uterine rupture is a catastrophic event. Maternal mortality ranges between 1 and 13 % and perinatal mortality between 74 and 92 % [6]. Rupture of unscarred pregnant uterus is a rare occurrence and it occurs in 1/5700 to 1/20,000 pregnancies [2], [3], [4], [5]. Its incidence is higher in developing countries. Rupture of unscarred uterus is associated higher likelihood of peripartum hysterectomy, haemorrhage and blood transfusions as compared to women who underwent rupture of scarred uterus [1]. Composite adverse perinatal outcomes are also more in rupture of scarred uterus as compared to rupture of unscarred uterus [1]. Occurrence of rupture of an unscarred uterus lays foundation for the importance of supervised labour and to maintain a high index of suspicion even in a prim gravida if she is showing classical signs of rupture uterus. Our case has been reported in accordance with the SCARE criteria [20].

## 2 Case presentation

A 22-year-old female primigravida, who was brought in ambulance to emergency room at 39 weeks period of gestation, at a tertiary care teaching hospital. She presented with complaint of pain abdomen. On examination she was found to be in latent labour. She was a Hindu by religion and a home maker. She was a booked and investigated case and had eight antenatal visits. All antenatal investigations were unremarkable. Mid trimester scan revealed a normally appearing anterior placenta. There was no history of intrauterine instrumentation or surgeries. During her stay in hospital, she had spontaneous progress of labour and there was no use of oxytocics for labour augmentation. She was admitted in the first stage of labour (1 cm dilated) and progressed to active labour spontaneously in 10 h. She became fully dilated after 4 h of active. Labour was well supervised by maintaining partograph and close foetal monitoring by intermittent auscultation.

In second stage, there was sudden cessation of contractions, loss of uterine contour and the foetal heart sound could not be localized. On per vaginum examination cervix was fully dilated and effaced with a loss of station. Patient was hemodynamically stable without any peritoneal signs. Urgent bedside ultrasound was done which revealed an empty enlarged uterus, a foetus in the abdominal cavity and foetal heart rate of 30 bpm. She was rushed to OT for an urgent laparotomy.

**Fig. 1:**
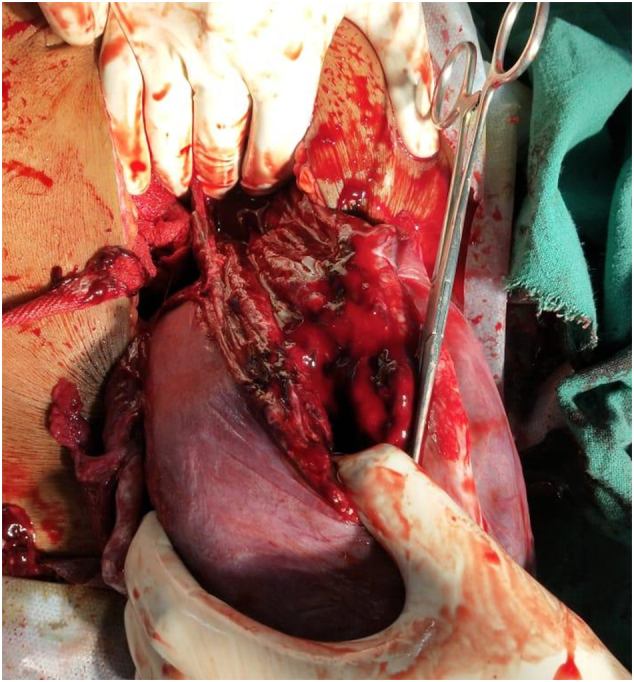
Rupture of left posterolateral aspect of the uterus.

**Fig. 2:**
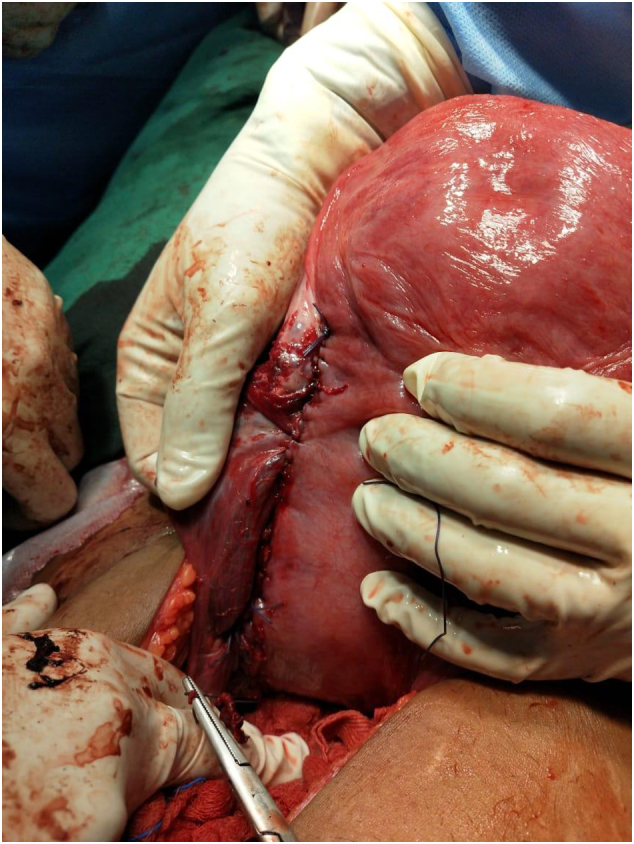
Rupture site repaired in two layers.

Midline Laparotomy revealed hemoperitoneum of 100 ml. Foetus and placenta were found outside the uterus in abdominal cavity. Uterus had no structural anomalies with a normal appearing placenta. A live male neonate weighing 2280 g was delivered. APGAR was 4, 8,8 at 1, 5 and 10 min respectively. Neonate was intubated after initial steps of resuscitation. On further inspection, a 10 cm tear found in left posterolateral aspect of uterus extending deep into the lower uterine segment. The tear extended into the left broad ligament. Bladder was mobilized. To counteract blood loss, we performed a left sided uterine artery ligation before repairing the tear. We were able to repair the tear in two layers. Intra operative blood loss was 1200 ml. Surgery was concluded by doing an extensive intraperitoneal lavage (Fig. 1, Fig. 2).

Patient had an uneventful post-operative period and did not require blood transfusion. She was discharged on day 7. She was advised early antenatal visit in subsequent pregnancy and delivery by elective caesarian section.

Her neonate developed grade 3 Hypoxemic ischaemic encephalopathy. After receiving assisted ventilation for a course of 3 days in Neonatal Intensive Care Unit, the neonate eventually died on day 7 of post-natal life.

## 3 Clinical discussion

Presence of a uterine scar is the key factor leading to rupture. Spontaneous rupture of unscarred uterus is a rare and is associated with trauma, grand multipara, unsupervised augmentation of labour, injudicious use of uterotonic agents, obstructed labour, Mullerian anomalies, obstetric manuevers (internal podalic version, breech extraction) and placenta percreta.

In our case, all these factors were ruled out. Ehler Danlos syndrome IV has been associated with uterine rupture but we didn't carry out the genetic testing. She did not have any relevant family history of connective tissue disorders. Patient was a primigravida who went in spontaneous labour without use of any oxytocics or labour inducting agents. There was no history of any kind of intrauterine instrumentation or manipulation in the past. She was a booked case and her antenatal ultrasounds ruled out possibility of Mullerian anomalies or abnormal placentation. Her labour was well supervised in a tertiary care centre. There was no undue prolongation of latent and active stage of labour. Active stage was well monitored with partograph, intermittent foetal auscultation and cardiotochography as per institutional protocol. In active labour, features of rupture were promptly detected and patient was taken for laparotomy after confirming the diagnosis on ultrasound. Due to early diagnosis and well-coordinated multidisciplinary approach, we were able to provide quick surgical intervention which is usually the key to successful management of uterine rupture [7]. We were able to prevent significant maternal morbidity in terms of excess blood loss, blood transfusion, post-operative sepsis and need for prolonged hospitalization. We were able to successfully repair the uterine rupture site, thus preserving her fertility and allowing her to reconsider child birth in future.

In literature search we could find 15 cases of spontaneous rupture in unscarred uterus (Table 1). Out of these 7 cases were prim gravidas [8], [9], [10], [13], [14], [16], [19]. In 6 cases, rupture occurred in primigravidas with no obvious risk factors [8], [9], [10], [14], [16], [19]. In most cases, rupture occurred before onset of labour and in only two cases, the woman was in labour when rupture uterus was diagnosed [16], [18].

**Table 1:** Case reports of uterine rupture in unscarred uteri.

Serial no	Case	Parity	Gestation	Labour status	Risk factors	Site of rupture
1.	Abbi 1997 [8]	Primi		Not in labour	None	
2.	Langton 1997 [9]	Primi	32 weeks	Not in labour	None	2 cm vertical tear above right uterosacral ligament
3.	Wang 1999 [10]	Prim	21 weeks	Not in labour	None	Cornual rupture
4.	Rana 2009 [11]	G3P2L2	32 weeks	Not in labour	Multipara	Right side of fundus 2 cm anterior to cornua
5.	Silva 2012 [12]	G2P1L1	32 weeks	Not in labour	Multipara	Left side of the anterior aspect of the uterus extending from the lower segment to the fundus.
6.	Sun 2012 [13]	G3P2L2	17 weeks	Not in labour	Multipara	Fundus
7.	Mitzutamari 2014	Primi	32 weeks	Not in labour	Arcuate uterus	Right cornua
8.	Manini 2016 [15]	G3P1L1A1	15 weeks	Not in labour	Multipara History of curettage	Fundus
9.	Mourad 2014 [16]	Prim	36 + 4 weeks	Early labour	None	Left anterior fundus to right corner of LUS
10.	Abdallah 2015 [17]	G3P1L1A1	28 weeks	Not in labour	History of curettage Multipara	Posterior wall of uterus
11.	Neilson 2017 [18]	G3P1L1A1	39 + 4	In labour	Multipara	
12.	Lotte 2017 [19]	Prim	31 + 2	Not in labour	None	In fundus near left tube insertion
13.	Maryam 2017 [22]	G2P1L1	12 weeks	No	Multipara	Posterior wall of uterus
14.	Theofanakis 2018 [21]	G2E1	16 weeks	No	• Previous history of right salpingectomy • Septate uterus	Right uterine horn
15.	Sun 2019	Prim	34 + 5 weeks	No	ART Multiple gestation Bilateral salpingectomy	Fundus

According to our best knowledge this is the 7th case of rupture in unscarred uterus, reported in a prim gravida without any obvious risk factors. In our case, the cause may be an underlying connective tissue disorder as other causes were ruled out. Although rupture was diagnosed in second stage, there were no features of uterine hyper stimulation, precipitate labour or obstructed labour.

## 4 Conclusion

Uterine rupture should always be considered in all cases if there is high clinical suspicion, even in primigravida. Early surgical intervention is a key to successful management.

## Provenance and peer review

Not commissioned, externally peer-reviewed.

## Ethical approval

None/exempted.

## Funding

No funding taken.

## Consent

Written informed consent was obtained from the patient for publication of this case report and accompanying images. A copy of written consent is available for review by the The Editor-in Chief of this journal on request.

## Author contribution

Dr. Mona Mishra: Study design, data collection and writing the paper.

Dr. Y. M Mala: Study design and writing.

## Guarantor

Dr. Y. M Mala, Professor.

## Research registration number

Not applicable.

## Declaration of competing interest

No conflict of interest.
